# Cross-Sectional Study of the Changes in Attitudes of Post-Acute Coronary Syndromes Patients Towards Remote Biosignal Monitoring an eHealth Support in a 5-Year Interval

**DOI:** 10.3390/jcm14176272

**Published:** 2025-09-05

**Authors:** Natalia Tsoumani, Iosif Klironomos, Margherita Antona, Nikos Kampanis, George E. Kochiadakis, Constantine Stephanidis, Spyridon Karageorgos, George Notas

**Affiliations:** 1 Institute of Applied Computational Mathematics, Foundation for Research and Technology Hellas, 70013 Heraklion, Greece; nataliatsoumani@hotmail.com (N.T.); kampanis@iacm.forth.gr (N.K.); 2 Department of Emergency Medicine, University of Crete School of Medicine, 71500 Heraklion, Greece; 3 Institute of Computer Science, Foundation for Research and Technology Hellas, 70013 Heraklion, Greece; iosif@ics.forth.gr (I.K.); antona@ics.forth.gr (M.A.); cs@ics.forth.gr (C.S.); 4 Department of Cardiology, University of Crete School of Medicine and University Hospital of Heraklion, 71500 Heraklion, Greece; kochiadg@uoc.gr; 5 Department of Computer Science, University of Crete, 71500 Heraklion, Greece; 6 First Department of Pediatrics, Aghia Sophia Children’s Hospital, National and Kapodistrian University of Athens, 11527 Athens, Greece; spiroskarageorgo@gmail.com; 7 Faculty of Medicine and Dentistry, Blizard Institute, Queen Mary University of London, London E1 2AT, UK

**Keywords:** acute coronary syndrome, patient acceptance of healthcare, mobile applications, telemedicine, privacy

## Abstract

**Background**: Mobile health (mHealth) applications have shown promise for the primary and secondary prevention of diseases in high-risk individuals. Implementing mHealth solutions for secondary prevention and early alert systems in patients with acute coronary syndrome (ACS) could have significant societal benefits. However, the attitudes of at-risk populations towards these technologies, including concerns about technological literacy and privacy, have not been thoroughly investigated. As technology incorporation expands, these issues are expected to change. This study aimed to evaluate the attitudes of post-ACS patients towards varying levels of intrusive mHealth applications and how these attitudes evolved over a five-year period. **Methods:** A cross-sectional study was carried out with two cohorts of post-ACS inpatients (110 patients each from 2014 and 2019), who were surveyed using a 39-item questionnaire assessing their technological literacy and opinions on support tools and intrusive technologies, such as wearables and GPS tracking. **Results:** The two cohorts exhibited stable demographic characteristics, but in 2019, participants showed higher technological literacy and increased engagement in travel and physical activities. Notably, there was a significant rise in hypertension, hyperlipidemia, and family history of Coronary Artery Disease (CAD) in the 2019 cohort. Acceptance of remote health monitoring improved significantly in 2019, influenced by technological literacy. **Conclusions:** Attitudes towards eHealth solutions and remote biosignal monitoring post-ACS may change over time with increased technological literacy. Future research should address patient-specific concerns that could affect the acceptance of new technological solutions to enhance post-ACS outcomes. Our findings emphasize the importance of improving technological literacy to boost the adoption and effectiveness of eHealth interventions.

## 1. Introduction

In Europe, more than one million people are admitted to hospitals each year with acute coronary syndromes (ACS), including acute myocardial infarction and unstable angina. Sudden deaths associated with early fatal arrhythmia and ventricular fibrillation are also significant concerns [1]. After hospital discharge, patients embark on a lifelong journey to mitigate the risks of coronary artery disease (CAD) mortality and morbidity, necessitating continuous intensive health monitoring. To address this need, various applications and electronic devices have been developed, marking the rise of e-health as a critical component of modern healthcare.

Over the past decade, the remote monitoring of patients outside traditional healthcare settings (e.g., hospitals, nursing homes, doctors’ offices) has been the focus of intense research [2]. Significant progress has been made in the Ambient Intelligence (AmI) and electronic health (eHealth) sectors toward developing such platforms [3]. Advances in telecommunications and the reduction in information transmission costs [4] are expected to further enhance home health monitoring solutions, which can be personalized to each patient’s needs. These systems are anticipated to reduce hospitalization duration [5], provide rapid EMS responses to acute events [6], help patients achieve healthy behaviors/goals [7], and ultimately improve patient quality of life [8].

Thus, ACS and CAD significantly burden societies and healthcare systems (GBD, 2017). Since more than half of the deaths post-MI occur within the first month [9], it is crucial to have a monitoring system that can quickly identify and activate EMS services and medical teams, potentially improving access to necessary healthcare services and protecting patients from significant morbidity and mortality. Furthermore, these patients are characterized by several risk factors that, if not properly managed, can lead to ACS relapses. AmI/eHealth platforms can assist patients in achieving health goals related to these risk factors, such as blood pressure and glucose control, increased physical activity, a healthy diet, and weight management.

Several software developers have designed applications aimed at the primary or secondary prevention of diseases like Chronic Obstructive Pulmonary Disease (COPD) and Cardiovascular Disease (CVD), focusing on promoting healthy behaviors [10]. The development of these systems is expected to reduce hospitalization durations, provide rapid Emergency Medical Services (EMS) responses to acute events, help patients achieve their health goals, and thus enhance their quality of life.

However, age, technological literacy, and cultural aspects can influence patients’ attitudes toward these monitoring/support platforms. There is a knowledge gap regarding patients’ attitudes toward using new technologies for remote health support, mainly when these technologies target diseases primarily affecting older and technologically less knowledgeable patients. Clinicians often assume that not all patients can manage modern eHealth platforms, but studies quantifying this issue are limited. Additionally, local and regional patient characteristics should be considered, as conclusions from urban area surveys may not apply to patients in rural areas where technological penetrance is generally lower.

The study aimed to explore the differences in attitudes, perceptions, and technological literacy of patients with recent ACS. It focused on the acceptance of recording biosignals and their remote monitoring by medical personnel over a 5-year interval in two cohorts. The study also examined the characteristics, personal attitudes, and technological literacy that influence patients’ acceptance of biosignal monitoring.

## 2. Materials and Methods

We performed a cross-sectional study with the same questionnaire in two different patient cohorts 5 years apart. Eligibility criteria included recent (within the last 15 days) diagnosis of an ACS and capacity to communicate and provide consent. Exclusion criteria included dementia or another disease that could impair judgment, inability to understand Greek, blindness, and deafness.

### 2.1. Study Sample

First Study Sample (April 2014–August 2014):

Patients diagnosed with ACS at the Cardiology Department of the University Hospital of Heraklion between April 2014 and August 2014 were invited to participate in an interview. ECG, troponin-I levels, and coronary angiography findings confirmed each patient’s ACS diagnosis. Among these patients, 63 (57.3%) had a stent placed during hospitalization. Out of 150 approached patients, 110 agreed to participate, resulting in a participation rate of 73.3%.

Second Study Sample (January 2019–June 2019):

Five years later, the same questionnaire was administered to a new cohort of patients admitted with ACS to the same Cardiology Department from January 2019 to June 2019. Similarly, ACS diagnoses were confirmed via ECG, troponin-I, and coronary angiography findings, with 55 patients (60.0%) receiving a stent. Of the 130 patients approached, 110 completed the interview, yielding a participation rate of 84.6%.

In both studies, the size was decided based on what the researchers considered a representative population sample that could allow the generalization of the findings and was comparable with similar published studies.

### 2.2. Questionnaire Description

The survey included 39 questions, divided into the following sections:

- Personal Data: Standard demographics such as age, sex, and activity frequency;
- Technological Literacy: Use of smartphones, internet services, etc.;
- Opinions and Perceptions about eHealth Services and Monitoring;
- Five questions addressed the inconvenience/annoyance of using a body-attached mobile recording device;
- Eight questions focused on the patient’s willingness to be observed for medical or activity monitoring.

Responses were measured on a four-point Likert scale from 0 (don’t know) to 3, ranging from minor inconvenience/annoyance or willingness (1) to refusal of observation/recording (3). The annoyance/inconvenience scale ranged from 0 to 15 (maximum annoyance), while the acceptance of observation scale ranged from 0 to 24 (maximum disagreement with observation). Cronbach’s alpha for internal consistency was 0.940 for annoyance/availability and 0.829 for the observation scale, indicating acceptable reliability. All questions were closed-ended.

Technological literacy was assessed using a dedicated section that used a four-point Likert scale (from 1 = good use to 4 = no use) to evaluate patients’ self-reported proficiency with a range of technologies, including tablets, old-type mobile phones, smartphones, computers, email, social media, and government-related e-services. For the multivariate regression analysis, we created a composite “technological literacy score” for each patient by summing the responses, resulting in a scale ranging from 7 to 28. The questions that assessed technological literacy also had a four-point Likert scale from 1 (good use) to 4 (no use), with a Cronbach’s alpha of 0.791, indicating acceptable reliability. The specific mHealth and biosignal monitoring technologies that were described in the questionnaire included (a) measuring vitals remotely, (b) the acceptance of wearable devices (shirts, watches, patches, etc.), (c) remote recording of daily activities and GPS position, (d) reminders for and monitoring of medication compliance, (e) chatbots, (f) home recording devices for emergency situations, (g) communication preferences, (h) exercise schedules via applications, (i) technology preferences (buttons, sizes, videos vs written instructions, etc.), and (j) appointment reminders.

### 2.3. Survey

Following ethics approval, trained health professionals (nurse, doctor, social worker) visited the cardiology department to evaluate all ACS patients against the inclusion criteria: adults admitted for ACS, not at risk during the interview, and able to understand Greek.

Participants who agreed to join the study signed a consent form and were informed they could withdraw anytime.

The study was conducted in Greek, with the original questionnaire included in the Supplemental Material.

The questionnaire was validated by assessing reliability (evaluation of random error, precision, reproducibility, repeatability, and consistency) and test–retest reliability. Internal consistency was examined using Cronbach’s alpha (0.87), and test–retest reliability was assessed through the Intraclass Correlation Coefficient (ICC) after a second completion of the questionnaire by 15 of the participants one week later. For this test–retest reliability process, the order of the questions was changed to ensure that participants would not remember their responses from the first completion of the questionnaire. Cronbach’s alpha was 0.87 and ICC was 0.84, suggesting reliability of the questionnaire.

### 2.4. Ethics

The study was approved by the University Hospital of Heraklion bioethics committee, protocol number 8172/11-6-2014 for the first cohort and protocol number 05/06-2-2019 for the second cohort. Researchers who were also health professionals conducted the interviews and the data collection. They provided additional information about the questions as needed and demonstrated smartphone or tablet functionalities to help patients respond accurately.

### 2.5. Statistical Methods

Continuous variables are expressed as mean ± SD, while counts and percentages are used for discrete variables. The Kolmogorov–Smirnov test was used to assess normality. Depending on the distribution, an independent samples t-test or Mann–Whitney test was used to compare two groups and a one-way ANOVA or Kruskal–Wallis test was used for more than two groups. Pearson’s chi-square test was applied to associate two discrete variables. Cronbach’s alpha assessed the internal consistency of questionnaire items. Mediation analysis was performed with the combination of linear regression function and the Sobel test (https://quantpsy.org/sobel/sobel.htm accessed on 22 August 2025). Statistical analyses were performed using IBM SPSS Statistics 26.0, with a significance level set at *p* = 0.05.

## 3. Results

Two cohorts of 120 participants each were evaluated five years apart. To address potential sources of bias, we compared the demographic characteristics of each dataset and their comparisons are presented in Table 1. The age distribution did not significantly differ between the two cohorts (*p* = 0.346). The most frequent age group in both cohorts was 56–65 years, with 31 participants (28.2%) in the 2014 cohort and 38 participants (34.5%) in the 2019 cohort. The mean age was 60.1 ± 13.7 years for the 2014 dataset and 62.5 ± 11.6 years for the 2019 dataset (*p* = 0.158). Regarding gender, males constituted the majority of post-ACS patients, with 81 males (73.6%) in the 2014 cohort and 89 males (80.9%) in the 2019 cohort. The gender distribution did not show a significant difference between the two periods. Regarding education level, university-educated participants were more prevalent in 2019, with 43 participants (39.1%) compared to 28 participants (35.5%) in 2014. However, this difference was not statistically significant (*p* = 0.070). Employment status also did not show any noticeable change, with non-working participants (housewives, retired, and unemployed) slightly increasing from 50 participants (45.5%) in 2014 to 60 participants (54.5%) in 2019 (NS). Living arrangements also remained relatively stable between the two cohorts. Overall, the demographic characteristics of the two cohorts indicate similar distributions across age, gender, education level, employment status, and living arrangements, with only minor variations that were not statistically significant. This consistency supports the comparability of the two groups in the study context.

Table 2 details the characteristics of the two cohorts’ hobbies and activities. In 2014, 39 participants (35.5%) reported having a hobby, compared to 46 participants (41.8%) in 2019. The distribution of hobbies and their frequency did not show significant differences between the two periods. Travel and trips were more frequently mentioned by participants in 2019, with 21 participants (19.1%) indicating they often engage in these activities, compared to 11 participants (10.0%) in 2014 (*p* = 0.010). Additionally, sports and workouts were more systematically practiced in 2019, with 36 participants (32.7%) engaging in these activities regularly, compared to 27 participants (24.5%) in 2014. Conversely, those not participating in sports or workouts were significantly fewer in 2019 (16.4%) compared to 2014 (36.4%). These findings suggest a trend towards increased engagement in travel and physical activities among the 2019 cohort, highlighting potential lifestyle shifts over the five years.

**Figure 1:**
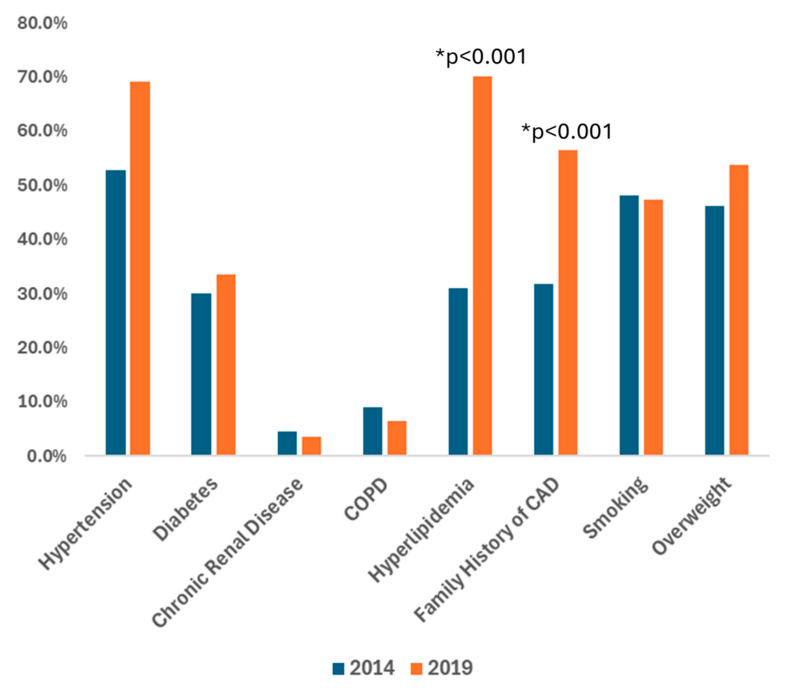
Characteristics of the medical history of participants in the 2014 and the 2019 cohorts. The technological literacy profile of the two cohorts demonstrated a significant increase in the use of mobile phones and self-reported proficiency in their use in 2019 compared to 2014. In 2019, 55.5% of participants reported regular use of mobile phones, up from 16.5% in 2014 (*p* < 0.001), and 55.0% rated their capacity to use them as “good,” compared to 26.4% in 2014 (*p* < 0.001). Additionally, the 2019 cohort showed a notable rise in self-reported “good” capacity for using email, with 40.9% of participants indicating proficiency, up from 20.9% in 2014 (*p* = 0.002). Moreover, the use of government-related e-services saw a substantial increase, from 17.3% in 2014 to 37.4% in 2019 (*p* = 0.012). These findings highlight a marked improvement in technological literacy over the five years. **COPD:** chronic obstructive pulmonary disease; CAD: Coronary Artery Disease.

Figure 1 presents the patient’s medical history for 2014 and 2019. Significant differences existed in the proportion of known cardiological risk factors between these two periods. Specifically, the 2019 cohort had higher incidences of hypertension, hyperlipidemia, and a family history of CAD. However, there were no significant differences between the two cohorts in the prevalence of diabetes, chronic kidney disease, smoking, obesity, and chronic obstructive pulmonary disease (COPD). This data indicates a notable increase in certain cardiovascular risk factors over the five years, underscoring the evolving health profile of the patient population.

Table 3 summarizes participants’ opinions on continuously recording vital signs at home during hospitalization. Demographic characteristics did not significantly affect patients’ views on remote recording. However, the 2019 cohort showed a considerably higher agreement with remote recording, with 93 participants (84.5%) in favor, compared to 66 participants (60.0%) in the 2014 cohort.

When multiple logistic regression was applied, using the acceptance of remote recording of vital signs as the dependent variable and a set of explanatory variables including cohort year (2014/2019), working status (employed/unemployed), educational level (primary/secondary/university), hobbies (no/yes), trips/travels (no/yes), and technological literacy score (range 7 to 28), it was found that the year of observation (cohort year) positively influenced opinions about remote health data recording (OR: 3.35, 95% CI: 1.75–6.43, *p* < 0.001). Technological literacy also had a positive effect (OR: 1.06, 95% CI: 1.00–1.11, *p* = 0.043). To examine whether higher education contributes to greater technological literacy, which in turn influences a patient’s willingness to accept remote monitoring, we conducted a mediation analysis. In this analysis, educational level served as the independent variable, technological literacy acted as the mediator, and acceptance of remote monitoring of vital signs was the dependent variable. Our findings indicated that the impact of higher education on the acceptance of remote monitoring was not mediated via an increase in technological literacy. This suggests that, between 2014 and 2019, technological literacy increased even in individuals without higher education. Detailed results of multiple logistic regression analyses can be found in the Supplementary Material (Table S1).

**Figure 2:**
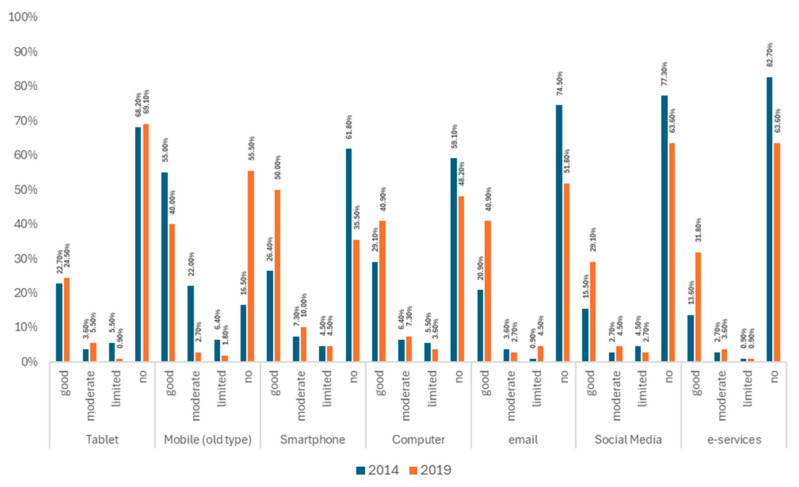
Technological literacy of participants in the 2014 and 2019 cohorts.

Table 4 presents the mean scores of patients’ opinions regarding monitoring for emergency health problems and vital signs, the inconvenience/annoyance of recording through a wearable device, and their technological literacy. The acceptance of biosignal observation had similar mean scores in both the 2014 and 2019 cohorts. Likewise, the inconvenience/annoyance scores were comparable between the 2014 and 2019 cohorts. However, technological literacy significantly differed between the two cohorts, with the 2019 cohort showing improved scores (mean of 19.4 ± 5.7) compared to the 2014 cohort (mean of 21.2 ± 6.7) (*p* = 0.026). Data on technological literacy between 2014 and 2021 are presented on Figure 2. Additional details, including frequencies of observational and recording items, can be found in the Supplementary Section (Tables S2 and S3).

## 4. Discussion

The American Heart Association highlights that health technology—including mobile apps, wearables, and telehealth—can facilitate self-monitoring, goal setting, and reminders, which are associated with improved adoption of healthful behaviors such as increased physical activity, dietary modification, and medication adherence in cardiovascular populations. However, the effectiveness of these interventions is contingent on patients’ digital literacy and ability to engage with these technologies, as limited technological literacy can be a barrier to both device acceptance and sustained behavior change [11,12]. Systematic reviews and clinical studies demonstrate that technology-based patient education (via text messaging, web platforms, and smartphone applications) leads to significant improvements in modifiable cardiovascular risk factors, including exercise, diet, blood pressure, and medication adherence among patients with coronary heart disease, provided that patients possess adequate eHealth literacy [13,14,15]. The American Heart Association further notes that digital literacy is a critical determinant of equitable access to these benefits, and interventions that address both health and digital literacy are necessary to maximize behavioral change and health outcomes [11,12].

Our study found significant consistency in demographic characteristics across two cohorts over five years, suggesting that age, gender, education level, employment status, and living arrangements remained stable. This demographic stability supports the validity of comparing data from 2014 and 2019, especially given that age is a known factor influencing fear and acceptance of technology [14,15,16,17]. This consistency provides a robust foundation for examining other variables and trends.

Interestingly, we observed notable differences in patients’ engagement in travel and physical activities, with the 2019 cohort showing increased involvement. This trend aligns with findings from other studies, such as a Canadian age-period-cohort analysis that reported more significant participation in physical activities in later generations [18]. Similarly, a Dutch research indicated increased physical activity in recent generations, though its impact on health indices remains unclear [19]. Despite suggestions that active travel can reduce morbidity and mortality from chronic diseases [20], our study did not find significant changes in ACS prevalence related to these activities, likely due to our limited sample size.

Our data revealed an increase in hypertension, hyperlipidemia, and family history of CAD in the 2019 cohort, indicating a worsening cardiovascular risk profile. This trend may reflect enhanced awareness, screening, and diagnosis efforts, consistent with US studies showing increased hypertension awareness and control from 1999 to 2016 [21]. Similarly, cholesterol screening and statin use have increased worldwide, which could explain the annual reduction observed in age-standardized death rates related to hyperlipidemia [22]. The rising family history of CAD in our cohort is particularly concerning due to its association with poor outcomes independent of other risk factors [23]. An apparent paradox emerges from our findings: the 2019 cohort displayed higher technological literacy and acceptance, yet also a higher prevalence of cardiovascular risk factors. It is critical to interpret this not as a failure of technology, but as a reflection of the timeline of health innovation. The increased prevalence likely signals improved diagnostic rates. The subsequent step—leveraging the newfound technological acceptance to improve the management and control of these diagnosed conditions—is the true challenge for clinical practice and the focus of future eHealth implementation studies. Our results establish a more receptive patient population, which is the foundational first step toward achieving this goal.

One of the most significant findings of our study is the increase in higher education levels and technological literacy in the 2019 cohort, which was also correlated with improvements in mobile phone use and proficiency and increased use of government-related e-services. This improvement is supported by numerous studies across various countries and age groups [24]. The rise in technological literacy significantly influences patients’ acceptance of remote monitoring. This effect is more substantial than the anticipated increase in technological literacy resulting from higher education among patients with CAD, as demonstrated by our mediation analysis. Instead, it seems to be a societal outcome related to improved access to these technologies. Enhanced digital literacy has been linked to better intergenerational integration [25], but varies by country context. For instance, the technological literacy of heart failure patients in Uganda differs from those in the USA, where electronic health records are more commonly used [26,27]. However, improved technological literacy can enhance patient engagement with digital health tools, boosting health literacy and transforming healthcare delivery and patient outcomes [28], while being scalable and effective for managing cardiovascular conditions in settings with limited resources [29]. Implementing solutions in developing countries faces unique challenges, including limited internet access, gaps in digital literacy, device availability, and data privacy concerns, particularly for populations with poor social determinants of health [12]. Research in Uganda and Tunisia shows the feasibility and positive patient acceptance of telehealth and digital platforms, but also highlights the need for further investigation into cost-effectiveness, workflow integration, and long-term outcomes [30,31].

Our 2014 cohort showed low acceptance of remote health monitoring, a sentiment that significantly improved in the 2019 cohort. This shift may be due to higher education levels, increased technological literacy, and societal advancements related to general technology acceptance. Studies have shown limited acceptance of eHealth among less technologically literate older adults, particularly those with age-prevalent conditions like cardiological issues [32]. Cultural characteristics and demographics significantly influence eHealth acceptance, particularly in heart failure patients [33]. Although we observed a trend toward higher acceptance of remote monitoring among younger patients, it was not statistically significant, likely due to our small sample size.

Technological literacy appears to be a critical factor in accepting remote biosignal monitoring and eHealth interventions. Studies indicate a positive association between technological and health literacy [34,35]. A systematic review found that less than half of remote monitoring trials evaluated patient perceptions, emphasizing the need for comprehensive patient attitude assessments in eHealth applications [36]. Cross-country analyses further reveal that IT literacy, rather than age, is the primary predictor of mHealth acceptance, underscoring the importance of user-friendly software for improving acceptance [37,38]. Our findings align with this notion, as demonstrated by our regression analysis.

Privacy and security concerns significantly influence the adoption of eHealth technologies and wearable devices. While recent privacy regulations like the General Data Protection Regulation (GDPR) in Europe aim to enhance data protection, our study revealed an unexpected finding: the pre-GDPR cohort expressed more skepticism about privacy issues than the post-GDPR group. Our findings emphasize that the successful development of healthcare applications requires a deep understanding of patients’ privacy concerns, particularly regarding wearable devices. These concerns encompass various aspects of data handling, including collection frequency, storage methods, and transmission protocols. A notable perception gap exists between healthcare providers and patients. While medical professionals and IT specialists view data collection as a health protection measure, patients often prioritize privacy risks over the potential health benefits of remote monitoring. Our research suggests that only healthcare institutions with exceptional reputations for reliability and trustworthiness can effectively overcome patients’ privacy concerns and encourage data sharing. This presents a significant challenge: as privacy concerns intensify in the digital healthcare landscape, they may impede the development and adoption of innovative eHealth technologies. This barrier could slow the advancement of digital healthcare solutions that could benefit patient care.

Our study has several limitations. It is a single-center pilot study, and the specific socio-economic and cultural characteristics of our cohorts may not apply to other populations. For example, there may have been regional differences in technology access that we did not adequately study. However, this highlights the necessity of personalized approaches in healthcare. While our cohorts showed no significant demographic or clinical differences over five years, differences in education and technological literacy were evident. This effect is a meaningful result, even for this small sample size. Although our cohorts were well-matched on key demographics, the study could have been further strengthened by a longitudinal follow-up of the initial cohort or the application of statistical techniques like propensity score matching to control for even minor baseline differences. Our analysis also did not account for potential shifts in standard-of-care treatment protocols or lifestyle counseling for ACS patients between the two study periods. Such changes could have independently influenced patient health awareness and, consequently, their attitudes towards health technology. The acceptance rates of biosignal monitoring in our cohorts should be compared cautiously with other studies, as many involve the general population. Although we focused on technological literacy, which was clearly correlated with age, socio-economic status did not seem to affect our results. While our findings demonstrate a positive shift in patient acceptance of eHealth, a crucial limitation is the absence of long-term clinical outcome data. Future prospective, longitudinal studies are imperative to determine whether higher acceptance and engagement with remote monitoring technologies translate into tangible clinical benefits, such as reduced hospital readmissions, lower mortality rates, and improved secondary prevention adherence in the post-ACS population. Such studies should correlate the level of technology adoption with hard clinical endpoints. Furthermore, our regression model did not include specific measures of patient health beliefs or nuanced social support structures, which are known to influence health behaviors and technology acceptance. Future research should incorporate validated scales for these psychosocial constructs to build a more comprehensive predictive model. A significant and insightful suggestion for future research is the application of more advanced statistical techniques, such as Latent Class Analysis, to move beyond population-level trends and identify distinct patient subgroups. While beyond the scope of the present study, an LCA approach would be invaluable for uncovering the nuanced interplay between technological literacy, health status, and personal attitudes. Based on our findings, we can hypothesize the emergence of several clinically relevant patient archetypes, which include (a) the ‘Eager Adopter’: Likely younger, with higher educational attainment, high technological literacy, and fewer privacy concerns, who are immediately ready for eHealth integration; (b) the ‘Willing but Wary’: Patients who trust the healthcare system and are willing to adopt new technologies but are hampered by lower technological literacy (ideal target for structured training and support programs); (c) the ‘Capable Skeptic’: Individuals who possess high technological literacy but have significant privacy and data-security concerns (interventions would need to focus on building trust through transparent data governance policies and enhanced security features); and (d) the ‘Digitally Excluded’: A group with low technological literacy and potentially limited access to technology, for whom current eHealth solutions may be unsuitable without significant family or caregiver support. Identifying the prevalence of these distinct subgroups within the post-ACS population is a critical next step. It would allow for a paradigm shift from a one-size-fits-all approach to a stratified model, where eHealth interventions are precisely tailored to the specific needs, skills, and concerns of each patient profile, thereby maximizing engagement and the potential for improved clinical outcomes.

A final limitation is the absence of a post-COVID-19 cohort. The COVID-19 pandemic acted as a catalyst for the adoption of digital health technologies globally and significantly influenced technological acceptance [39,40]. Such a cohort could transform our findings into a three-point longitudinal societal assessment. Nonetheless, our study provides valuable baseline data for pre-pandemic populations, useful for future large-scale research on the pandemic’s effects.

While our findings are specific to the post-ACS population, they underscore a broader principle: patient acceptance is context-dependent. Future research should investigate the unique attitudes and concerns of patients with other cardiovascular conditions, such as chronic heart failure or arrhythmias, as the specific biosignals, monitoring devices, and data transmission requirements for these populations differ substantially.

## 5. Conclusions

Our study suggests that attitudes towards eHealth solutions and remote monitoring of biosignals after an ACS may evolve over time with improved technological literacy. Our study briefly addresses privacy issues, but further research is needed to explore patients’ data-security concerns using mixed methods. Conducting qualitative interviews could reveal the specific fears patients have, which would help in designing more transparent and trustworthy eHealth systems. These insights emphasize the need to improve technological literacy to boost the adoption and effectiveness of eHealth interventions.

## Figures and Tables

**Table 1:** Demographic characteristics of post-ACS patients in the two cohorts.

		Cohort				
		2014		2019		
		N	%	N	%	P
**Age groups**	**≤45**	14	12.7	8	7.3	0.346
	**46–55**	29	26.4	21	19.1	
	**56–65**	31	28.2	38	34.5	
	**66–75**	19	17.3	25	22.7	
	**76+**	17	15.5	18	16.4	
**Sex**	**Woman**	29	26.4	21	19.1	0.198
	**Man**	81	73.6	89	80.9	
**Educational Level**	**Primary**	47	42.7	34	30.9	0.070
	**Secondary**	35	31.8	33	30.0	
	**University+**	28	25.5	43	39.1	
**Working**	**No**	60	54.5	50	45.5	0.178
	**Yes**	50	45.5	60	54.5	
**Living in**	**House**	78	70.9	69	62.7	0.198
	**Apartment**	32	29.1	41	37.3	
**Living with other**	**Yes**	98	89.1	93	84.5	0.319

N = number of cases, P = *p*-value.

**Table 2:** Hobbies and activities of post-ACS patients (f = frequency).

		Cohort				
		2014		2019		
		N	%	N	%	P
**Hobbies**	**Yes**	39	35.5	46	41.8	0.332
**Hobbies,** f	**Never**	71	64.5	64	58.2	0.798
	**Rarely**	8	7.3	9	8.2	
	**Monthly**	2	1.8	3	2.7	
	**1–2 times/week**	15	13.6	21	19.1	
	**Everyday**	14	12.7	13	11.8	
**Travels/Trips**	**Yes**	75	68.2	86	78.2	0.094
**Travels/Trips, f**	**Never**	35	31.8	24	21.8	0.011
	**Rarely**	26	23.6	39	35.5	
	**Yearly**	21	19.1	20	18.2	
	**Every 2–3 years**	17	15.5	6	5.5	
	**Often**	11	10.0	21	19.1	
**Sports**	**Yes**	70	63.6	92	83.6	0.001
**Sports, f**	**Systematic**	27	24.5	36	32.7	0.010
	**Periodically**	19	17.3	23	20.9	
	**Rarely**	24	21.8	33	30.0	
	**Never**	40	36.4	18	16.4	

N = number of cases, P = *p*-value.

**Table 3:** Opinions for acceptance of continuous recording of the vital signs of patients near hospitalization.

		Remotely Recording of Vital Signs				P
		Yes		No		
		N	%	N	%	
**Cohort**	**2014**	66	60.0	44	40.0	**<0.001**
	**2019**	93	84.5	17	15.5	
**Sex**	**Woman**	34	68.0	16	32.0	0.443
	**Man**	125	73.5	45	26.5	
**Age groups**	**≤45**	19	86.4	3	13.6	0.158
	**46–55**	39	78.0	11	22.0	
	**56–65**	51	73.9	18	26.1	
	**66–75**	29	65.9	15	34.1	
	**76+**	21	60.0	14	40.0	
**Working**	**No**	73	66.4	37	33.6	0.050
	**Yes**	86	78.2	24	21.8	
**Educational Level**	**Primary**	51	63.0	30	37.0	0.062
	**Secondary**	53	77.9	15	22.1	
	**University+**	55	77.5	16	22.5	
**Hobbies**	**Yes**	67	42.1	18	29.5	0.085
**Trips/Travels**	**Yes**	122	75.8	37	62.7	0.055

N = number of cases, P = *p*-value.

**Table 4:** Comparison of patients’ opinion scores regarding the observation of their biosignal and the annoyance/inconvenience associated with wearable devices.

Score	Cohort	Mean	SD	p*
**Observation of biosignals**	2014	6.5	3.9	0.969
	2019	6.5	2.9	(0.857)
**Annoyance/Inconvenience** **of recording**	2014	14.5	5.9	0.503
	2019	14.1	4.2	(0.244)
**Technological Literacy**	2014	21.2	6.7	0.026
	2019	19.4	5.7	(0.002)

p* value in parentheses refers to the Mann–Whitney test.

## Data Availability

The data of the study can be provided freely to researchers upon request.

## Supplementary Materials

The following supporting information can be downloaded at: https://www.mdpi.com/article/10.3390/jcm14176272/s1, Table S1: Multiple logistic regression results for prediction of acceptance of personal vital signs observation from cohort year, educational level, working status, hobbies, travels/trips and technological literacy as explanatory variables using enter and backward selection; Table S2: Opinions about inconvenience/annoyance of recording of medical data; Table S3: Opinions about continuous observation of medical data.
