# Supplementary material for: Cross-Sectional Study of the Changes in Attitudes of Post-Acute Coronary Syndromes Patients Towards Remote Biosignal Monitoring an eHealth Support in a 5-Year Interval
Source: J Clin Med. 2025 Sep 5;14(17):6272. doi: 10.3390/jcm14176272 (PMC12429493; doi:10.3390/jcm14176272)
Supplement: Supplementary file 1 [file jcm-14-06272-s001.zip › jcm-3815415-supplementary.pdf]

Supplementary Material

Table S1. Multiple logistic regression results for prediction of acceptance of personal vital signs observation from cohort year, educational level, working status, hobbies, travels/trips and technological literacy as explanatory variables using enter and backward selection.

		OR	95% CI		p
			LB	UB	
**Enter**	**Year 2019**	**3,39**	**1,75**	**6,56**	**<0,001**
	**Education=Secondary**	0,60	0,27	1,31	0,198
	**Education=University**	0,90	0,38	2,15	0,815
	**Working=Yes**	0,81	0,39	1,71	0,587
	**Hobbies=Yes**	1,27	0,63	2,58	0,505
	**Travel-Trips=Yes**	1,32	0,64	2,70	0,454
	**Technological Literacy (High to Low)**	1,03	0,96	1,10	0,370
**Backward**	**Year 2019**	3,35	1,75	6,43	**<0,001**
	**Technological Literacy (High to Low)**	1,06	1,00	1,11	**0,043**

Table S2. Opinions about inconvenience/annoyance of recording of medical data.

		Disturb me a little		Disturb me a lot		Can not Stand		Don't know	
**Cohort**	**Recording**	n	%	n	%	n	%	N	%
**2014**	**No automatic**	19	17.3%	59	53.6%	14	12.7%	18	16.4%
	**Automatic**	18	16.4%	63	57.3%	13	11.8%	16	14.5%
	**wearable (clothe)**	16	14.5%	58	52.7%	19	17.3%	17	15.5%
	**Wearable**	14	12.7%	64	58.2%	15	13.6%	17	15.5%
	**patch**	16	14.5%	56	50.9%	22	20.0%	16	14.5%
**2019**	**No automatic**	26	23.6%	72	65.5%	7	6.4%	5	4.5%
	**Automatic**	26	23.6%	72	65.5%	7	6.4%	5	4.5%
	**wearable (clothe)**	24	21.8%	71	64.5%	8	7.3%	7	6.4%
	**Wearable**	23	20.9%	74	67.3%	5	4.5%	8	7.3%

patch	25	22.7%	71	64.5%	8	7.3%	6	5.5%

Table S3. Opinions about continuous observation of medical data

		Don't know		Yes, no restrictions		Yes, with Restrictrions		No	
**Cohort**	**Observation**	**n**	**%**	**n**	**%**	**n**	**%**	**n**	**%**
**2014**	**with camera**	13	11.8%	17	15.5%	23	20.9%	57	51.8%
	**Mobile**	14	12.7%	37	33.6%	20	18.2%	39	35.5%
	**drugs use**	10	9.1%	44	40.0%	15	13.6%	41	37.3%
	**weight watch**	9	8.2%	44	40.0%	13	11.8%	44	40.0%
	**data collection**	11	10.0%	37	33.6%	17	15.5%	45	40.9%
	**environmetal devices**	11	10.0%	40	36.4%	19	17.3%	40	36.4%
	**messages**	12	10.9%	63	57.3%	24	21.8%	11	10.0%
**2019**	**with camera**	3	2.7%	9	8.2%	14	12.7%	84	76.4%
	**Mobile**	5	4.5%	53	48.2%	33	30.0%	19	17.3%
	**drugs use**	5	4.5%	62	56.4%	30	27.3%	13	11.8%
	**weight watch**	6	5.5%	66	60.0%	28	25.5%	10	9.1%
	**data collection**	7	6.4%	67	60.9%	25	22.7%	11	10.0%
	**environmetal devices**	9	8.2%	51	46.4%	26	23.6%	24	21.8%
	**messages**	1	0.9%	58	52.7%	27	24.5%	24	21.8%
